# Diet-derived nutrient patterns and components of metabolic syndrome: a cross-sectional community- based study

**DOI:** 10.1186/s12902-020-0547-0

**Published:** 2020-05-19

**Authors:** Mahdi Vajdi, Mahdieh Abbasalizad Farhangi, Leila Nikniaz

**Affiliations:** 1 Nutrition Research Center, Tabriz University of Medical Sciences, Tabriz, Iran; 2 Drug Applied Research Center, Tabriz University of Medical Sciences, Tabriz, Iran; 3 Tabriz Health Services Management Research Center, Health Management and Safety Promotion Research Institute, Tabriz University of Medical Sciences, Tabriz, Iran

**Keywords:** Obesity, Nutrient pattern, Factor analysis, Metabolic syndrome, Iranian population

## Abstract

**Background:**

Metabolic syndrome (MetS) is one of the main public health problems worldwide. Although some relations between dietary intakes and MetS have been found, few studies have focused on association between dietary nutrients interactions and the risk of the MetS and its components. The aim of the present study was to examine the association between nutrient patterns and MetS and its components among Iranian adult population.

**Methods:**

A total of 588 subjects (aged 18–64 years, 271 males and 317 females) enrolled in the cross sectional study. Dietary consumption was evaluated using an 80-item food frequency questionnaire (FFQ). Biochemical assessments including fasting blood sugar (FBS), aspartate aminotransferase (AST), alanine amino transferase (ALT) and serum lipids were performed by enzymatic methods. Nutrient patterns were obtained by factor analysis procedure using principal component method. Multinational logistic regression analysis was used to evaluate the association between nutrient patterns and MetS and its components.

**Results:**

Three nutrient patterns explaining 53.66% of the variance in dietary nutrients intake, were recognized in the current study. Animal-sourced nutrient pattern was significantly associated with the higher odds of MetS and high triglyceride (TG) levels. Plant-sourced nutrient pattern (high intake of fiber, carbohydrate, vitamins B_6_, B_3_, C, B_1_, E, D, magnesium, potassium, and linoleic acid) was significantly associated with lower risk of MetS and lower blood pressure (*p* < 0.05). Third nutrient pattern (mixed-source) was significantly related to higher risk of MetS, high waist circumference (WC) and high systolic blood pressure (SBP).

**Conclusion:**

This present study confirms the important effect of nutrients and their patterns on MetS risk. Our results suggest that adherence to the nutrient pattern rich in fiber, carbohydrate, vitamins D, B_6_, B_3_, C, B_1_, E, magnesium, potassium, linoleic acid, and docosahexaenoic acid (DHA) is associated with a lower risk of MetS, while animal- and mixed-sourced nutrient patterns are positively associated with greater odds of MetS; However, further longitudinal and interventional studies are required to make a clear conclusion.

## Background

The metabolic syndrome (MetS) is characterized by a clustering of cardiovascular risk factors, including central obesity, elevated blood pressure, dyslipidemia (high triglyceride and low high-density lipoprotein cholesterol (HDL-C)) and fasting hyperglycemia [1]. The prevalence of MetS has been increasing worldwide and it has become a main public health problem in numerous countries.

The MetS prevalence varies from 11.6 to 62.5% around the worldwide [2] and according to a recent systematic review and meta-analysis the prevalence of MetS in Iran was 35 and 24% in women and men, respectively [3].

The etiology of MetS is complex and involves the interactions between metabolic, environmental and genetic factors [4]. In addition to ethnic predisposition, physical activity, smoking, stress and body mass index (BMI), diet, as a major lifestyle factor, may be strongly associated with the development of MetS [5]. Dietary intervention is a fundamental strategy for the prevention and treatment of MetS [6]. In recent decades, several studies have evaluated the effect of dietary patterns and isolated foods on development of MetS [7, 8] and some results have proposed that consumption of vegetables and fruits is linked with a reduced risk of MetS [9, 10]. Fruits and vegetables are main contributors of magnesium, vitamin C, potassium, and vitamin A, and they may potentially decrease the risk of chronic life-threatening diseases [11].

There are several methods to examine the associations of dietary intake and food groups with MetS in epidemiologic studies [12, 13]. It has previously been proposed that dietary patterns may provide valuable perception of diet-disease associations [14] and maybe more predictive of chronic disease risk than the consumption of single foods or nutrients [15]. Several researches on dietary patterns have shown that adherence to prudent, Dietary Approach to Stop Hypertension (DASH) diet, Mediterranean diet are associated with lower risk of MetS [16–19]. In contrast, a meta-analysis by Rodríguez-Monforte M et al. in 2017 revealed that unhealthy dietary patterns (i.e. Western diet) were positively associated with MetS [20].

Since individuals do not consume isolated nutrients but rather eat meals consisting of a variety of foods with multiple nutrients, the traditional method of assessing the diet and disease relationship, which focuses on highly correlated nutrients separately, maybe inappropriate for taking into account cumulative synergistic or interactive effects on the circulating levels, metabolism, bioavailability, and excretion of nutrients [14]. Thus, assessing the combined effect of nutrients through creating nutrient patterns could be an appropriate alternative method for assessing the effects of diet on diseases [21]. The nutrient pattern method is a combination of multiple nutrients and may provide more information about probable underlying mechanisms, interactions and synergic effects of nutrients [15]. Several studies have found the inverse association between MetS and single nutrients such as vitamin D [22], calcium [23], vitamin C [24], carotene [25], and potassium [26]. There are limited studies conducted in Iran that evaluated the association between the nutrient patterns and MetS and only Khayyatzadeh et al. [27] have previously evaluated this relationship in Mashhad, Northeast Iran. They reported that a nutrient pattern which mostly characterized by dietary maltose, glucose, carbohydrate, sucrose, protein, starch, and fructose, was related to a higher risk of MetS in both sexes. Until now, there is no study in the literature investigating the relationship between nutrient patterns and MetS in northwest of Iran. So, the aim of this study was to recognize any nutrient patterns and subsequently, to examine the relationship between the nutrient patterns and components of MetS in northwest of Iran.

## Methods

### Study population

The data for this cross-sectional study were taken from major lifestyle promotion project (LPP) in East Azerbaijan, Iran, aimed to explore the prevalence of non-communicable diseases and the associated risk factors. Probability proportional to size multistage stratified cluster sampling was used as a sampling method. The exact method of sampling is described in our previous study [28]. Briefly, 150 clusters were selected and 590 participants from 150 clusters were enrolled. Incomplete questionnaires were excluded and the final sample subjected to analysis was 588 individuals. People were included in the current study if their original nationality was Iranian and aged between 18 and 64 years. Individuals with physical disability, severe chronic illness requiring bed rest, active liver injury, mental disability, and pregnant women were excluded. The Ethics Committee of Tabriz University of Medical Sciences approved the current study (Registration number: IR.TBZMED.REC.1398.077) and all individuals signed the written informed consent before participating in this cross-sectional study. The work is obtained from M.S. thesis of Mahdi Vajdi.

### General characteristics and anthropometric measurements

The anthropometric, demographic and dietary information were collected by expert health care professionals. The demographic information included age, education, physical activity, marital and smoking status. The socio-demographic characteristics and smoking status were collected through a direct interview using a questionnaire and physical activity level was evaluated using the Official Persian short form of international physical activity questionnaire (IPAQ-SF) [29]. Body weight was measured using a digital scale (Seca, Dubai, United Arab Emirates) while subjects minimally clothed without shoes and height was measured to the nearest 0.5 cm with a stadiometer. Body mass index (BMI) was then calculated by weight (kg) divided by height squared (m^2^). Blood pressure was measured with a standard mercury sphygmomanometer with a standard cuff twice in the same arm in sitting position. Waist circumference (WC) was measured in horizontal plane, midway between the iliac crest and the rib cage with a measuring tape in centimeter. Waist to hip ratio (WHR) was calculated by waist circumference divided by hip circumference.

### Dietary assessment

Trained dietitians collected dietary information over the preceding 1 year through an 80-item quantitative food frequency questionnaire (FFQ), which was developed and validated for LPP study and reference portions were defined based on the most reported portion sizes in the 24-h recall [30]. Subjects were asked to report their frequency and the amount of intake for each food item on a daily, weekly, monthly or yearly basis. Reported frequency of each food item, were then converted to daily gram intake. In addition to the daily energy, 40 nutrients intake for each subject were computed using the US Department of Agriculture’s (USDA) and Nutrients Composition of Iranian Foods. In the present study, we used protein, carbohydrate, fat, total dietary fiber, cholesterol, saturated fatty acids (SFAs), monounsaturated fatty acids (MUFAs), polyunsaturated fatty acids (PUFAs), vitamin A, vitamin E, vitamin D, vitamin K, thiamin, pantothenic acid, vitamin B12, biotin, riboflavin, niacin, pyridoxin, vitamin C, folate, Alpha tocopherol, linolenic acid, linoleic acid, DHA, eicosapentaenoic acid (EPA), potassium, chromium, magnesium, sodium, iron, phosphorus, calcium, manganese, copper, fluoride, selenium, zinc, sugar and caffeine to identify nutrient patterns.

### Biochemical assessment

About 10 ml blood sample was taken from each participant after an overnight fast. All the samples were immediately centrifuged at 2000 rpm for 10 min at room temperature. Fasting blood sugar (FBS) and Lipid profile [triglyceride (TG), High-Density Lipoprotein-Cholesterol (HDL-C) and total cholesterol (TC)] were evaluated using enzymatic colorimetric technique (Pars Azmoon commercial kit, Tehran, Iran) and Low-Density Lipoprotein-Cholesterol (LDL-C) was measured by Friedewald eq. [31]. Alanine aminotransferase (ALT) and aspartate transaminase (AST) were assessed using the ultraviolet method [32]. Chemiluminescent immunoassay technology was used for the quantitative determination of serum 25 (OH) D (25 OH Vitamin D Total Assay, DiaSorin, Saluggio, Italy).

### Definition of the metabolic syndrome

Participants were categorized as having MetS according to the internationally accepted criteria of NCEPATP III [33], if they have three or more of the following characteristics: TG ≥ 150 mg/dl or current use of medicine for dyslipidemia; WC ≥ 88 cm in women and ≥ 102 cm in men; diastolic blood pressure (DBP) ≥85 mmHg or systolic blood pressure (SBP) ≥ 130 mmHg or using antihypertensive medication; HDL-C < 50 mg/dl in women and < 40 mg/dl in men or current use of medicine for dyslipidemia and FBS ≥100 mg/dl or on medication treatment for hyperglycemia.

### Statistical analyses

All analyses were carried out using the Statistical Package for Social Sciences (SPSS, version 21, Chicago, IL, USA). The principal component method for factor analysis was employed to determine major nutrient patterns (called factors) based on the forty predefined nutrients and three explainable factors were kept. The reliability of the factor analysis was tested by Kaiser-Meyer-Olkin (KMO) test and orthogonal Varimax rotation was carried out to clarify the interpretability and decrease the correlation between the factors. The number of factors was determined by considering scree plot, eigenvalues > 1.00 and the interpretability of the factors. Factor loadings of the nutrients in each nutrient pattern were computed. Positive loading in a factor shows a direct relationship with the factor, while a negative loading shows that the nutrient is inversely relationship with the factor. We calculated the factor score for each participant and nutrient pattern by summing up intakes of nutrients weighted by their factor loadings. For each nutrient pattern, participants were grouped into four categories according to quartiles of factor scores. Continuous variables are presented as the means ± standard deviations, and categorical variables are expressed as the number (%). Categorical and continuous variables were compared across quartiles of nutrient pattern scores using analysis of x^2^ and analysis of variance (ANOVA) tests, respectively. To find the association between nutrient patterns and odds of MetS and its components, multinational logistic regression so was used in different models. Model 1 was adjusted for sex, age, smoking, physical activity, education levels and energy intake. The first quartile was used as a reference for calculating the odds ratios (OR).

## Result

The mean age of the participants was 42.26 ± 11.97 years and the mean BMI was 27.37 ± 4.81 kg/m^2^. About 46.1% of the population was male. The prevalence of high DBP, SBP, TG, WC, FBS and low HDL were 26.7, 29.4, 38.8, 60.4, 43.9 and 13.6% respectively while total prevalence of MetS was 39.3%. In the current study three major nutrient patterns based on 40 food components were extracted: animal-sourced pattern characterized by high intake of saturated fatty acid (SFA), fat, protein, sugar, vitamins B_5_, B_12,_ B_2_, A, K, biotin, phosphorus, cholesterol, selenium, sodium, polyunsaturated fatty acids (PUFA), calcium and zinc; plant-sourced pattern with high consumption of fiber, carbohydrate, vitamins B_6_, B_3_, C, B_1_, E, D, magnesium, potassium, linoleic acid, and DHA and mixed-source nutrient pattern including fluoride, manganese, caffeine and folate.

The identified nutrient patterns described 53.66% of the total variance in dietary nutrient intake (Table 1). The participants’ characteristics across quartiles of nutrient patterns are presented in Table 2. Across different quartiles of animal-sourced pattern, those in top quartiles had significantly higher weight, smoking habits, energy and macronutrient intake compared to the participants in the first quartile (*p* < 0.05). Within the plant-sourced pattern, those in higher quartiles had significantly higher weight, energy and macronutrient intake compared to the participants in the lower quartiles (*p* < 0.05) and those in lower quartiles had significantly higher age and higher prevalence of hypertension than compared to the participants in the higher quartiles (*p* < 0.05). Participants in higher quartiles of mixed-source nutrient pattern had significantly higher BMI, energy and carbohydrate intake compared to the participants in the bottom quartile (p < 0.05).

**Table 1:** Factor loading matrix for the nutrients representing the three major nutrient patterns

Nutrients	Animal sourced	Plant sourced	Mixed-source
Fatty acid- saturated	**0.951**	0.196	_
Fat	**0.932**	0.253	_
Protein	**0.920**	0.345	_
Vitamin B_5_	**0.908**	0.361	0.125
Vitamin B_12_	**0.901**	0.258	_
Phosphorus	**0.878**	0.358	_
Cholesterol	**0.828**	0.222	_
Selenium	**0.812**	0.380	_
Sodium	**0.810**	0.195	_
Vitamin B_2_	**0.784**	0.453	0.300
Vitamin K	**0.729**	0.292	_
Calcium	**0.704**	0.171	_
Zinc	**0.610**	0.253	_
Sugar	**0.563**	0.147	0.128
Vitamin A	**0.472**	0.367	_
PUFA	**0.333**	0.324	_
Biotin	**0.112**	_	_
Alpha tocopherol	_	_	_
Chromium	_	_	_
MUFA	_	_	_
Copper	_	_	_
Linolenic acid	_	_	_
Total fiber	_	**0.891**	_
Vitamin B_6_	0.497	**0.786**	_
Carbohydrate	0.207	**0.785**	0.176
Vitamin B_3_	0.538	**0.709**	_
Vitamin C	_	**0.673**	_
Magnesium	0.453	**0.670**	0.497
Vitamin D	_	**0.633**	_
Vitamin B_1_	0.153	**0.619**	_
Potassium	0.327	**0.549**	0.443
Vitamin E	0.138	**0.468**	_
Linoleic acid	0.262	**0.293**	_
DHA		**0.101**	
Iron	_	_	_
Fluoride	_	_	0.982
Manganese	_	0.124	0.980
Caffeine	_	_	0.976
Folate	0.447	0.559	0.603
EPA	_	_	_
Percentage of Variance explained	27.60	16.23	9.82

Loading values of 0.10 or greater are indicated by bold value. Abbreviations: *MUFA* monounsaturated fatty acid, *PUFA* polyunsaturated fatty acids, *DHA* docosahexaenoic acid, *EPA* eicosapentaenoic acid

**Table 2:** Baseline characteristics of study population stratified by nutrient patterns

Variable	Animal sourced				p	Plant sourced				p	Mixed-source				p
	Q1	Q2	Q3	Q4		Q1	Q2	Q3	Q4		Q1	Q2	Q3	Q4	
Age (years)	41.53 ± 12.28	43.18 ± 12.44	42.25 ± 11.45	42.08 ± 11.64	0.69	44.34 ± 11.95	40.31 ± 11.36	42.60 ± 12.28	41.78 ± 11.92	**0.03**	40.52 ± 12.27	41.87 ± 12.09	42.93 ± 11.73	43.72 ± 11.54	0.11
Gender (male)	44(29.9)	59(40.1)	76(51.7)	92(62.6)	**0.01**	62(42.2)	68(46.3)	71(48.6)	70(47.3)	0.71	77(52)	60(40.3)	67(46.9)	67(45.3)	0.24
BMI (kg/m^2^)	27.63 ± 4.36	27.30 ± 4.45	27.34 ± 5.14	27.21 ± 5.27	0.89	27.02 ± 4.61	27.12 ± 4.70	27.58 ± 4.60	27.78 ± 5.32	0.49	26.80 ± 5.03	26.90 ± 4.63	27.28 ± 4.67	28.50 ± 4.77	**0.01**
Weight (kg)	72.52 ± 12.81	71.78 ± 13.12	73.67 ± 11.41	75.78 ± 14.54	**0.04**	71.03 ± 11.76	73.41 ± 13.90	74.76 ± 13.81	74.67 ± 12.46	**0.04**	72.75 ± 14.32	72.36 ± 12.46	73.33 ± 13.50	75.40 ± 11.81	0.20
Hypertension	17(11.6)	23(15.8)	17(11.6)	18(12.2)	0.66	27(18.4)	11(7.5)	21(14.4)	16(10.9)	**0.03**	14(9.5)	17(11.4)	25(17.5)	19(12.8)	0.21
Current smoker 11(7.6)		16(11.2)	22(15.2)	28(19.3)	**0.02**	26(17.9)	21(14.5)	15(10.4)	15(10.5)	0.18	13(9.1)	18(12.2)	19(13.5)	27(18.6)	0.11
**Physical activity**					0.21					0.56					0.75
Low	38(25.9)	43(29.7)	51(34.7)	37(25.2)		40(27.2)	35(24)	44(30.1)	50(34)		39(26.4)	43(28.9)	42(29.8)	45(30.4)	
Medium	48(32.7)	44(30.3)	47(32)	61(41.5)		55(37.4)	50(34.2)	48(32.9)	47(32)		58(39.2)	45(30.2)	49(34.8)	48(32.4)	
High	61(41.5)	58(40)	49(33.3)	49(33.3)		52(35.4)	61(41.8)	54(37)	50(34)		51(34.5)	61(40.9)	50(35.5)	55(37.2)	
**Marital status**					0.83					0.63					0.49
Married	137(93.2)	132(90.4)	136(92.5)	135(91.8)		135(91.1)	132(89.8)	135(92.5)	138(93.9)		134(91.2)	134(89.9)	132(92.3)	140(94.6)	
**Education**					0.40					**0.01**					**0.04**
Illiterate	24(16.3)	27(18.5)	23(15.6)	16(10.9)		36(24.5)	18(12.2)	20(13.7)	16(10.9)		11(7.5)	27(18.1)	28(9.6)	24(16.2)	
≤ High school/ diploma 96(65.3)		100(68.5)	102(69.4)	112(76.2)		97(66)	104(70.7)	101(69.2)	108(73.5)		106(72.1)	101(67.8)	97(67.8)	106(71.6)	
≥ College degree 27(18.4)		19(13)	22(15)	19(12.9)		14(9.5)	25(17)	25(17.1)	23(15.6)		30(20.4)	21(14.1)	18(12.6)	18(12.2)	
Energy (Kcal)	2134 ± 729	2773 ± 629	3490 ± 605	5158 ± 1558	**0.01**	2604 ± 1121	3120 ± 1290	3556 ± 1173	4263 ± 1741	**0.01**	3519 ± 1427	3105 ± 1317	3370 ± 1504	3548 ± 1634	**0.03**
Protein (g)	94 ± 32	131 ± 27	175 ± 32	278 ± 83	**0.01**	133 ± 64	155 ± 71	176 ± 69	214 ± 105	**0.01**	173 ± 83	158 ± 75	172 ± 92	175 ± 87	0.27
Carbohydrate(g) 196 ± 92		189 ± 80	204 ± 76	242 ± 91	**0.01**	130 ± 32	178 ± 45	217 ± 48	306 ± 92	**0.01**	217 ± 90	187 ± 69	198 ± 84	229 ± 98	**0.01**
Fat(g)	115 ± 38	170 ± 33	223 ± 33	358 ± 107	**0.01**	176 ± 87	206 ± 98	225 ± 92	259 ± 136	**0.01**	223 ± 103	199 ± 95	220 ± 117	223 ± 119	0.17

*P*-value obtained using one-way ANOVA for continuous variables and Chi-square test for categorical variables. Categorical and continuous variables data are presented as number (percent) and mean (SD). Bold values indicate P < 0.05 as the level of significance. *BMI* body mass index

Biochemical and anthropometric characteristics of the study population across quartiles of nutrient pattern are presented in Table 3. We found that participants in the higher quartiles of the animal-sourced pattern had significantly higher DBP, TG, ferritin, and AST and the individuals in the second quartile had a higher HDL-C (*p* = 0.001) compared to first quartiles (*p* < 0.05). Within the plant-sourced pattern, participants in the higher quartile had significantly lower WC, WHR, SBP, FBS and LDL-C than those in the first quartile (*p* < 0.05). Participants in higher quartiles of mixed-source nutrient pattern had significantly higher WC and SBP B compared to the participants in the bottom quartile (p < 0.05).

**Table 3:** Anthropometric and biochemical measures across quartiles of nutrient patterns

Variable	Animal sourced				P	Plant sourced					Mixed-source				P
	Q1	Q2	Q3	Q4		Q1	Q2	Q3	Q4		Q1	Q2	Q3	Q4	
**WC (cm)**	91.48 ± 11.87	92.86 ± 13.83	92.43 ± 15.47	93.07 ± 13.01	0.77	95.70 ± 15.34	92.17 ± 12.88	92.45 ± 12.56	90.57 ± 13.29	**0.04**	88.26 ± 15.79	92.15 ± 11.61	94.06 ± 10.90	95.27 ± 15.53	**0.01**
**WHR**	0.87 ± 0.08	0.89 ± 0.10	0.89 ± 0.17	0.89 ± 0.08	0.21	0.92 ± 0.18	0.88 ± 0.08	0.88 ± 0.08	0.88 ± 0.09	**0.01**	0.87 ± 0.11	0.88 ± 0.07	0.90 ± 0.06	0.90 ± 0.17	0.34
**SBP (mmHg)**	120.72 ± 16.85	118.59 ± 17.5	120.27 ± 17.05	122.04 ± 20.38	0.45	123.82 ± 19.99	122.35 ± 18.35	121.25 ± 17.4	117.27 ± 10.99	**0.04**	117.66 ± 15.73	119.56 ± 15.01	124.06 ± 21.55	121.39 ± 18.89	**0.04**
**DBP (mmHg)**	74.32 ± 13.47	80.31 ± 12.31	80.61 ± 11.11	80.61 ± ±12.30	**0.01**	80.04 ± 12.89	78.91 ± 14.40	77.47 ± 10.38	77.91 ± 11.25	0.31	77.14 ± 11.64	78.32 ± 11.08	79.64 ± 13.41	79.17 ± 13.08	0.35
**FBS (mg/dl)**	88.00 ± 20.36	87.12 ± 15.36	89.01 ± 23.23	90.15 ± 32.09	0.74	92.65 ± 26.87	86.76 ± 20.81	86.54 ± 25.33	85.83 ± 22.22	**0.02**	87.39 ± 22.18	87.45 ± 19.57	91.11 ± 27.69	88.38 ± 24.67	0.53
**TG (mg/dl)**	152.75 ± 92.49	154.27 ± 79.48	156.83 ± 117.90	169.09 ± 101.78	**0.04**	163.65 ± 92.16	162.72 ± 107.21	160.0 ± 100.98	157.34 ± 96.67	0.95	165.67 ± 105.8	161.85 ± 88.36	161.20 ± 105.12	154.86 ± 96.14	0.84
**TC (mg/dl)**	176.90 ± 35.39	179.60 ± 49.4	180.20 ± 37.72	183.34 ± 39.03	0.62	185.67 ± 42.33	181.87 ± 39.88	177.00 ± 36.2	175.38 ± 42.71	0.14	179.37 ± 39.17	182.14 ± 39.4	180.93 ± 36.8	177.36 ± 46.17	0.78
**LDL**-C **(mg/dl)** 88.93 ± 26.71		91.34 ± 31.34	92.82 ± 29.14	93.96 ± 32.53	0.54	96.33 ± 33.76	93.38 ± 28.59	89.43 ± 30.21	87.62 ± 25.97	**0.04**	91.35 ± 26.26	90.88 ± 30.13	92.61 ± 30.80	91.93 ± 32.25	0.96
**HDL**-C **(mg/dl)** 44.98 ± 9.96		45.00 ± 9.72	43.56 ± 10.55	42.98 ± 9.96	**0.01**	44.02 ± 11.75	43.39 ± 10.03	43.45 ± 9.41	44.12 ± 9.53	0.90	43.36 ± 10.09	43.38 ± 10.33	44.05 ± 9.90	44.16 ± 10.54	0.87
**Vitamin D (ng/ml)** 22.57 ± 19.10		22.26 ± 16.58	23.31 ± 22.01	24.03 ± 27.95	0.79	23.94 ± 18.15	21.19 ± 25.46	22.70 ± 18.94	24.42 ± 24.35	0.39	23.84 ± 27.76	24.65 ± 19.29	23.77 ± 23.74	22.04 ± 14.75	0.61
**Ferritin**	70.35 ± 67.14	75.99 ± 72.07	87.29 ± 74.70	99.48 ± 71.49	**0.004**	84.50 ± 74.79	81.41 ± 72.03	81.31 ± 65.13	85.04 ± 76.82	0.96	84.95 ± 72.70	75.96 ± 62.70	81.03 ± 70.90	90.38 ± 80.89	0.40
**ALT (IU/l)**	17.92 ± 9.42	17.47 ± 8.51	18.98 ± 9.76	19.54 ± 10.16	0.19	17.67 ± 10.13	18.01 ± 9.40	18.75 ± 8.61	19.59 ± 9.83	0.36	18.11 ± 7.61	17.50 ± 8.49	19.94 ± 10.52	18.25 ± 10.84	0.14
**AST (IU/l)**	20.23 ± 6.10	21.00 ± 6.35	20.76 ± 5.58	22.26 ± 6.84	**0.04**	21.03 ± 6.66	21.37 ± 6.66	21.37 ± 6.48	21.45 ± 6.77	0.60	21.25 ± 5.83	20.81 ± 5.58	21.57 ± 6.88	20.72 ± 6.75	0.65

P-value for continuous variables was compared across quartiles of nutrient pattern scores using analysis of variance (ANCOVA) tests adjusting for age, BMI and energy intake. Continuous variables are presented as the means ± standard deviations. Bold values indicate P < 0.05 as the level of significance. *Abbreviations*: *WC* waist circumference, *WHR* waist hip ratio, *TG* triglyceride*, TC* total cholesterol, *AST* aspartate aminotransferase, *ALT* alanine aminotransferase, *HDL-C* high-density lipoprotein cholesterol, *FBS* fasting blood sugar, *SBP* systolic blood pressure, *DBP* diastolic blood pressure

**Fig. 1:**
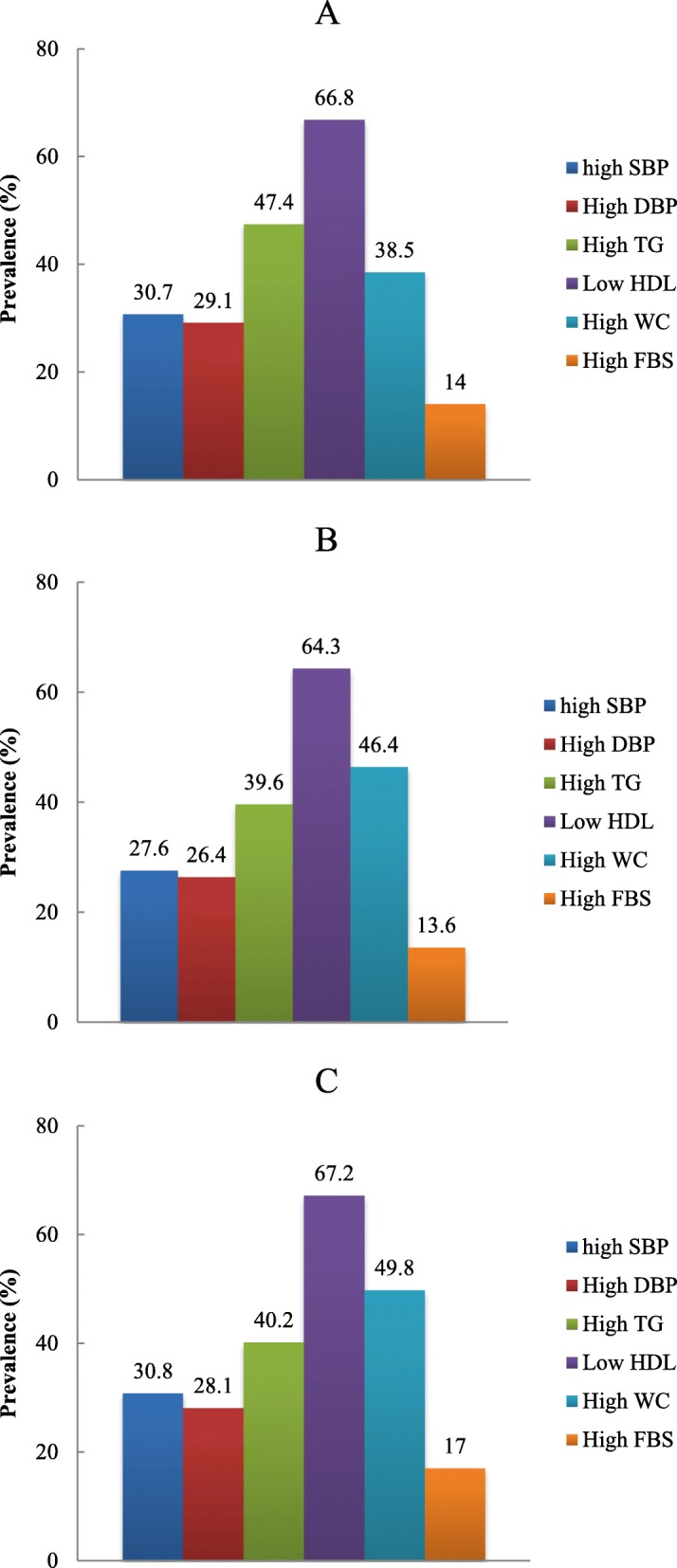
Prevalence of components of metabolic syndrome across animal-sourced nutrient pattern (**a**), plant-sourced nutrient pattern (**b**) and mixed-source nutrient pattern (**c**). WC, waist circumference; TG, triglyceride; HDL-C, high-density lipoprotein cholesterol; FBS, fasting blood sugar; SBP, systolic blood pressure; DBP, diastolic blood pressure

Multivariable-adjusted Odd’s ratio (OR) and confidence interval (CI) for MetS and its components across quartiles of dietary nutrient patterns are shown in Table 4. In an unadjusted model, participants in the top quartile of the animal-sourced pattern were more likely to have 2.35-fold higher odds for MetS compared with those in the first quartile (OR: 2.35, 95%CI: 1.11–5.92) and those in the fourth quartile of the animal-sourced pattern tended to have 1.36-fold higher odds for higher TG compared with those in the first quartile (OR: 1.36, 95% CI: 1.03–1.78). However, this relationship was no longer significant after controlling for sex, age, smoking status, education level and energy intake. In an unadjusted model, participants in the top quartile of the plant-sourced pattern were 50% less likely to have MetS compared with those in the first quartile (OR: 0.50, 95% CI: 0.28–0.87). However, this relationship was no longer significant after adjustment for confounders. Also, in an unadjusted model, significant inverse associations were noted between second quartile of the plant-sourced nutrient pattern and high SBP (OR: 0.46, 95% CI: 0.27–0.78) and high DBP (OR: 0.45, 95% CI: 0.27–0.77). Both in crude and adjusted regression analyses, participants in the third quartile of the mixed-source nutrient pattern were 71% more likely to have MetS (crude OR: 1.71 and adjusted OR:1.77) and tended to have 2.32-fold higher odds for higher SBP compared with those in the first quartile (OR: 2.32, 95% CI: 1.34–4.00). In the case of WC, those in the higher quartile of mixed-source nutrient pattern had significantly higher odds for higher WC compared with those in the lowest quartile (OR: 2.51, 95% CI: 1.55–4.06). Figure 1 presents the prevalence of components of MetS across three nutrient patterns. As shown in this Figure, having low serum HDL-C and high blood sugar had the highest and lowest prevalence among metabolic disorders of all of nutrient patterns. No difference between three nutrient patterns regarding the prevalence of metabolic disorders was reported.

**Table 4:** Odd’s ratio (OR) and confidence interval (CI) for MetS and its components according to quartiles (Q) of nutrient patterns

Variable	Animal sourced				P	Plant sourced					Mixed-source				P
	Q1	Q2	Q3	Q4		Q1	Q2	Q3	Q4		Q1	Q2	Q3	Q4	
**MetS**^A^															
**Crude**	1 (Ref.)	1.15(0.66–2.00)	1.29(0.67–2.49)	2.57(1.11–5.92)	**0.02**	1 (Ref.)	0.65(0.39–1.08)	0.74 (0.44–1.24)	0.50(0.28–0.87)	**0.01**	1 (Ref.)	1.19 (0.71–2.00)	1.71(1.02–2.85)	1.38(0.82–2.30)	**0.04**
Adjusted*	1 (Ref.)	1.09(0.67–1.76)	0.98(0.59–1.59)	1.39(0.85–2.27)	0.18	1 (Ref.)	0.64(0.39–1.06)	0.77(0.47–1.25)	0.63(0.38–1.03)	0.06	1 (Ref.)	1.19(0.72–1.97)	1.77(1.07–2.93)	1.41(0.85–2.33)	**0.02**
**High WC**															
**Crude**	1 (Ref.)	0.95(0.60–1.52)	0.76(0.48–1.22)	0.69(0.43–1.10)	0.12	1 (Ref.)	0.91(0.57–1.45)	1.16 (0.73–1.86)	1.06(0.66–1.68)	0.80	1 (Ref.)	2.10(1.29–3.40)	2.01(1.23–3.26)	2.51(1.55–4.06)	**0.001**
Adjusted*	1 (Ref.)	0.86(0.47–1.59)	0.82(0.40–1.69)	0.82(0.32–2.07)	0.67	1 (Ref.)	1.29(0.73–2.30)	1.64(0.91–2.96)	1.35(0.72–2.50)	0.34	1 (Ref.)	1.95(1.11–3.44)	1.95(1.10–3.45)	2.44(1.38–4.31)	**0.002**
**Low HDL-C**															
**Crude**	1 (Ref.)	0.85(0.51–1.42)	0.73(0.44–1.22)	1.01(0.61–1.69)	0.94	1 (Ref.)	1.30(0.77–2.19)	1.05(0.64–1.74)	0.92(0.56–1.52)	0.76	1 (Ref.)	1.15(0.69–1.92)	1.18(0.71–1.97)	1.15(0.69–1.92)	0.56
Adjusted*	1(Ref.)	0.99(0.56–1.71)	0.89(0.45–1.76)	1.25(0.53–2.97)	0.59	1 (Ref.)	1.41(0.82–2.44)	1.07(0.62–1.84)	0.81(0.45–1.43)	0.46	1 (Ref.)	1.13(0.66–1.91)	1.22(0.72–2.07)	1.09(0.64–1.85)	0.74
**High TG**															
**Crude**	1 (Ref.)	1.01(0.61–1.66)	1.26(0.95–1.68)	1.36(1.03–1.78)	**0.03**	1 (Ref.)	1.27(0.50–1.32)	1.23(0.53–1.38)	1.04(0.54–1.44)	0.41	1 (Ref.)	1.06(0.52–1.38)	1.10(0.42–1.13)	1.21(0.48–1.28)	0.69
Adjusted*	1 (Ref.)	1.06(0.53–1.65)	1.28(0.90–1.96)	1.39(1.48–2.60)	0.78	1 (Ref.)	1.35(0.43–1.33)	1.28(0.48–1.37)	1.17(0.54–1.52)	0.34	1 (Ref.)	1.09(0.36–1.03)	1.19(0.50–1.38)	1.28(0.42–1.17)	0.06
**Hyperglycemia**															
**Crude** 1(Ref.)		0.70(0.35–1.41)	0.83(0.42–1.63)	1.03(0.54–1.95)	0.91	1 (Ref.)	0.72(0.36–1.44)	0.70(0.35–1.40)	1.17(0.62–2.21)	0.61	1 (Ref.)	0.87(0.41–1.85)	1.70(0.86–3.34)	1.43(0.72–2.86)	0.30
Adjusted* 1 (Ref.)		0.67(0.33–1.71)	1.12(0.44–2.86)	1.24(0.38–3.98)	0.71	1 (Ref.)	0.80(0.38–1.67)	0.80(0.38–1.68)	1.31(0.62–2.76)	0.47	1 (Ref.)	0.86(0.39–1.85)	1.51(0.75–3.05)	1.41(0.69–2.88)	0.33
**High SBP**															
**Crude** 1(Ref.)		0.95(0.58–1-60)	0.90(0.54–1.50)	0.89(0.54–1.48)	0.66	1 (Ref.)	0.46(0.27–0.78)	0.72(0.44–1.190)	0.67(0.41–1.11)	**0.004**	1 (Ref.)	1.77(1.03–3.05)	2.32(1.34–4.00)	1.98(1.36–3.34)	**0.002**
Adjusted* 1(Ref.)		0.76(0.41–1.43)	0.86(0.42–1.79)	1.08(0.43–2.70)	0.85	1 (Ref.)	0.57(0.32–1.02)	0.81(0.46–1.44)	0.75(0.40–1.38)	0.36	1 (Ref.)	1.81(0.10–3.30)	2.30(1.27–4.19)	1.80(0.99–3.27)	**0.006**
**High DBP**															
1 (Ref**.) Crude**		1.07(0.63–1.81)	1.15(0.68–1.95)	1.26(0.75–2.13)	0.37	1 (Ref.)	0.45(0.27–0.77)	0.67(0.40–1.11)	0.54(0.32–0.90)	**0.02**	1 (Ref.)	0.98(0.57–1.69)	1.49(0.87–2.54)	1.43(0.84–2.42)	0.17
Adjusted*	1 (Ref.)	0.99(0.53–1.84)	1.39(0.67–2.86)	1.86(0.74–4.62)	0.18	1 (Ref.)	0.52(0.29–0.91)	0.69(0.39–1.21)	0.52(0.28–0.96)	**0.03**	1 (Ref.)	0.93(0.52–1.67)	1.37(0.78–2.43)	1.29(0.74–2.27)	0.36

The multivariate multinomial logistic regression was used for estimation of ORs and confidence interval (CI). Bold Values indicate P < 0.05 as the level of significance. *Adjusted for sex, age, smoking, physical activity, education levels and energy intake. ^**(A)**^ Defined as the presence of at least three of the following components: TG ≥150 mg/dl; WC ≥ 88 cm in women and ≥ 102 cm in men; DBP ≥85 mmHg or SBP ≥ 130 mmHg; HDL-C < 50 mg/dl in women and < 40 mg/dl in men and fasting glucose ≥100 mg/dl. *Abbreviations*: *MetS* metabolic syndrome, *TG* triglyceride*, WC* waist circumference, *HDL-C* high-density lipoprotein cholesterol, *SBP*, systolic blood pressure, *D*

*BP* diastolic blood pressure

## Discussion

Our study is the first to examine the association between nutrient patterns and MetS and its components in north-west Iran. Considering the recent increase in MetS prevalence in Iran, there was a terrible need to search for moderating factors. Although relations between nutrients and risk of multiple chronic conditions have received increased interest in recent years but a few studies are available on the association of nutrient patterns with risk of non-communicable diseases [8, 11]. Assessing nutrient patterns give us a snapshot of all dietary nutrients with all of their relations, which can be very important. Only one study evaluating the relationship between nutrient patterns and MetS in Iranian population. They reported that Western pattern (consisting of maltose, glucose, carbohydrate, sucrose, protein, starch, and fructose) was related to a higher risk of MetS in both sexes [27]. Our findings revealed three main nutrient patterns in East-Azerbaijan population: animal-sourced, plant sourced and mixed source nutrient pattern. We found that adherence to the “animal-source” and “mixed source” patterns were positively associated with MetS whereas plant source nutrient pattern was inversely associated the risk of MetS. Nutrients included in nutrient pattern analyses are very diverse between different studies. In this current study, we tried to include a maximum number of MetS-associated nutrients and bioactive food compounds (40 in total).

In this cross-sectional study, we found a significant positive association between animal-sourced nutrient pattern (consisting of SFA, fat, protein, sugar, vitamins B_5_, B_12,_ B_2_, A, K, biotin, phosphorus, cholesterol, selenium, sodium, PUFA, calcium and zinc) and odds of having MetS and high TG in study population. We also found a higher level of serum TG, AST, ferritin and SBP at the highest quartile of the animal-sourced nutrient pattern.

The relationship between isolated dietary consumptions of some nutrients in this nutrient pattern with MetS has been reported previously. Dietary intakes of SFA, sugar and cholesterol have been positively associated with MetS in previous studies [34, 35]. In contrast, some other nutrients in this pattern, such as vitamin C, selenium and zinc, have been inversely associated with MetS [36, 37]. In our study, SFA and PUFA, part of this nutrient pattern, were positively associated with risk of MetS but another study by Brady et al. [38] showed that there is no association between different dietary consumptions of omega-6 PUFAs and omega-3 PUFAs and the odds of MetS. Our results support the idea that a higher level of SFAs raises the risk of MetS.

Sugar and protein were also highly represented in animal-sourced nutrient pattern. In our study, protein was positively associated with MetS risk and high TG levels. In contrast to our results, several authors indicated that intake of protein-rich meals has some favorable effects such as regulation of appetite and increased thermogenesis which improves weight management and consequently decrease the risk of MetS and its components [39, 40]. A cohort study by Schulze et al. [41] demonstrated that higher intake of sugar-sweetened drinks may result in type 2 diabetes and MetS. Although there is some disagreement for some nutrients such as calcium and vitamin A, which is in agreement with our results, a cohort study conducted in France by Samara et al. [42] showed a positive and negative association for calcium levels with TG and HDL-c, respectively and Beydoun MA et al. [24] reported that vitamin A had a positive association with MetS and insulin resistance.

The existence of fat and sugar in this nutrient pattern may have resulted in a MetS-inducing effect; thus, it seems that sugar rise the odds of MetS even when coingested with nutrients that may contribute to protect against MetS. Therefore, the interaction of nutrients can affect their impact on MetS, and understanding these interactions in diets appears important.

In our study, plant-sourced nutrient pattern, which was positively correlated with total fiber, carbohydrate, vitamins D, B_6_, B_3_, C, B_1_, E, magnesium, potassium, linoleic acid, and DHA, was negatively related to the prevalence of MetS, high SBP and high DBP. Also, those in the top quartile of the plant-sourced nutrient pattern had lower SBP, WC, WHR, LDL-C and FBS levels than participants in the lowest quartile. Dietary intakes of magnesium, fiber, vitamin D and potassium have been inversely associated with MetS and obesity in previous studies [43–45]. Some other nutrients in this pattern, such as vitamin B_3_ [46] and B_1_ [47] have been positively associated with obesity. B-vitamins may lead to increased appetite; therefore, their long-term intake may trigger unnecessary energy consumption and weight gain [48].

Possible mechanisms may be through improvement of glucose and insulin metabolism by vitamins (E, B_6_, B_1_) and magnesium [49]; enhancement of endothelial function by vitamins (B_3_ and E) and magnesium [50]; reduced oxidative stress and increased HDL-C by magnesium and vitamins (B_3_ and E) [51] and reduction in blood pressure by magnesium and vitamins (B_3_, B_1_) [50]. Magnesium acts as a cofactor for several enzymes and plays an important role in the insulin and glucose metabolism [49]. A recent meta-analysis of six cross-sectional studies presented that dietary magnesium consumption is inversely related to the prevalence of MetS [52].

Among dietary factors, fiber consumption could play an important role in the control of MetS. In line with our study, a cross-sectional study conducted in Japan showed an inverse relationship between MetS and potassium and dietary fiber [43]. It is well known that increased potassium consumption decreases blood pressure by neutralizing the effects of sodium [53]. A recent systematic review and meta-analysis reported that high dietary potassium consumption was related to lower odds of MetS [45] also another study showed a negative relationship between serum potassium level and the prevalence of MetS [26]. Contrary to our results, Iwasaki et al. [43] concluded that vitamin E positively related to the odds of MetS, and high blood pressure but Wei et al. [37] did not detect a significant relationship between the consumption of vitamins (E and A) and the development of MetS. It is possible that synergistic influence of fiber, magnesium, potassium and other nutrients in this pattern cause the significant effect of fiber on decreasing the risk of MetS.

Also, our finding revealed that the mixed-source nutrient pattern (high intake of fluoride, manganese, caffeine and folate) was positively related to MetS, high WC and SBP. Several prior animal studies have proposed that fluoride may affect glucose metabolism and insulin sensitivity [54, 55]. Another cross-sectional study of 346 adults in northwestern Iran showed no relationship of fluoride in drinking water with WC, WHR and BMI [56]. The mechanisms by which fluoride interferes with MetS are unclear: nevertheless, fluoride may induce inflammation [57] and oxidative stress [58], all of which have been known to play an important role in obesity, hyperglycemia, dyslipidemia, and insulin resistance. Altogether, these risk factors significantly contribute to cardiovascular disease risk [59]. A study in Chinese adults over 18 years reported that higher manganese consumption was related to a reduced risk of the MetS in men (OR = 0.62) but positively related to low HDL-C among both men and women [60]. Previous animal studies have shown that manganese consumption might decrease abdominal fat deposition by reducing glycerol in adipose tissue [61], malate dehydrogenase and fatty acid synthase activities in the liver [62], also decrease TC, HDL-C and LDL-C because manganese may be an important component of the lipoprotein structure [63].

Caffeine, part of nutrient pattern 3, was positively associated with MetS risk. In line with the results, Stutz et al. in a cross sectional study, reported that coffee consumption was related to higher risk of MetS in population of subjects with type 1 diabetes [64] but in a cross-sectional study by Wilsgaard et al. [65] coffee intake was not significantly associated with incidence of MetS. Contrary to our results, Hino et al. demonstrated that coffee intake has beneficial effects on SBP, DBP, FBS, TG levels, and WC [66]. Therefore, the interaction of nutrients can affect their impact on MetS, and understanding these interactions in diets appears important. In the current study, extracted nutrient patterns were different from those reported for African [67] and Chinese populations [68].

The discrepancy between the results of the studies may be due to the different characteristics of the study populations, included nutrients, and also the area where the study was carried out. Significant protective effect was detected with plant-sourced nutrient pattern high in total fiber, carbohydrate, vitamins D, B_6_, B_3_, C, B_1_, E, magnesium, potassium, linoleic acid, and DHA among nutrient patterns found in current study. Nutrient patterns via taking into account the mixture of several nutrients, their interaction and the synergistic effect can be a strong forecaster of risk MetS. The major strengths of our study are the relatively large sample size, population based design and use of validated questionnaires. The present study has some limitations. As this is a cross-sectional observation, no causal associations can be determined. Since nutrient consumption was evaluated using an FFQ, some amount of measurement error is unavoidable.

## Conclusion

This present study confirms the important effect of nutrients on MetS risk. Our results suggest that adherence to the nutrient pattern rich in fiber, carbohydrate, vitamins D, B_6_, B_3_, C, B_1_, E, magnesium, potassium, linoleic acid, and DHA is associated with a lower risk of MetS, while animal- and mixed-sourced nutrient patterns are positively associated with greater odds of MetS; However, further longitudinal and interventional studies are required to make a clear conclusion.

## Data Availability

All of the data are available with reasonable request from the corresponding author.

## Abbreviations

MetS: Metabolic syndrome
PA: Physical activity
PCA: Principal Components Analysis
BMI: Body mass index
CVD: Cardiovascular disease
WC: Aist circumference
TG: Triglyceride
TC: Total cholesterol
HDL-C: High-density lipoprotein cholesterol
FBS: Fasting blood sugar
SBP: Systolic blood pressure
DBP: Diastolic blood pressure
FFQ: Food frequency questionnaire
CI: Confidence interval
IPAQ: International physical activity questionnaire
MET: Metabolic equivalent
SD: Standard Deviations
WHR: Waist hip ratio
